# Low Zinc, Copper, and Manganese Intake is Associated with Depression and Anxiety Symptoms in the Japanese Working Population: Findings from the Eating Habit and Well-Being Study

**DOI:** 10.3390/nu11040847

**Published:** 2019-04-15

**Authors:** Mieko Nakamura, Ayako Miura, Tomomi Nagahata, Yosuke Shibata, Eisaku Okada, Toshiyuki Ojima

**Affiliations:** 1 Department of Community Health and Preventive Medicine, Hamamatsu University School of Medicine, Hamamatsu 431-3192, Japan; shibata@hama-med.ac.jp (Y.S.); eisaku@hama-med.ac.jp (E.O.); ojima@hama-med.ac.jp (T.O.); 2 Department of Health and Nutrition, Faculty of Health Proportional Sciences, Tokoha University, Hamamatsu 431-2102, Japan; miura@hm.tokoha-u.ac.jp; 3 Department of Nutrition, School of Health and Nutrition, Tokaigakuen University, Nagoya 468-8514, Japan; nagaha-t@tokaigakuen-u.ac.jp

**Keywords:** K6, psychological distress, micronutrient, mineral, trace elements

## Abstract

Epidemiological studies have suggested that there is an association between diet and mental health. The aim of this study was to investigate the association between the intake of six minerals and mental disorders in a cross-sectional study. We used data from the Eating Habit and Well-being study in Japanese workers. Kessler’s six-item psychological distress scale was used to detect mental disorders, with a cut-off score of 12/13, and a validated food frequency questionnaire was used to estimate dietary mineral intake. A total of 2089 participants with no history of depression were included. The prevalence of mental disorders was 6.9%. The lowest quartiles of zinc, copper, and manganese intakes were associated with mental disorders, whereas the lowest quartiles of calcium, magnesium, and iron intake were not associated with mental disorders. Combination analysis of high (≥median) or low (<median) intake of zinc, copper, and manganese showed that low zinc and low copper intake, even with low or high manganese intake (odds ratio (OR), 2.71, 95% confidence interval (CI), 1.29–5.73, and OR, 3.06, 95% CI, 1.41–6.61, respectively) showed a higher OR than that of high zinc, high copper, and high manganese intake. Further studies are required to investigate the impact of dietary mineral intake on mental health.

## 1. Introduction

Mental and substance use disorders accounted for 22.9% of the non-fatal burden of disease in 2010, and represent the leading causes of disease worldwide [1]. Depressive disorders contribute most to the burden of disease, followed by anxiety disorders, drug use disorders, and alcohol disorders [1]. The working generation are at high risk of developing mental disorders [2]. The World Mental Health Japan Survey 2002–2005 reported that approximately 9% of workers had some mental disorder over the last 12 months, and the 30-day prevalence of major depressive disorder or any mood disorder was significantly associated with a decrease in work performance [3]. Thus, the Japanese government enforced the Stress Check Program, which screens psychosocial stress in the workplace in order to improve workers’ mental health, based on the Industrial Safety and Health Law of 2015 [4]. However, prevention strategies are still desirable to counter this burden.

Lifestyle factors including diet, sleep, and exercise are thought to represent significant mediating factors underlying the pathophysiological mechanisms associated with mental disorders [5]. An increasing number of epidemiological studies have suggested an association between diet and mental health, and propose that dietary intervention is important for the prevention and treatment of this modifiable risk factor [6,7]. Owing to the great burden of mental disorders, small improvements in diet may be greatly beneficial to mental health and well-being at the population level [7].

A previous meta-analysis found an inverse association between healthy dietary patterns and depression, and an insignificant positive association between the Western diet and depression [8]. A healthy dietary pattern includes a high intake of fruit, vegetables, poultry, fish, and whole grains, and a reduced intake of fat products. From the viewpoint of nutrients, associations between n-3 polyunsaturated fatty acids (PUFAs), folate, other B vitamins, and depression were most frequently investigated; however, many of the results have been controversial [9]. Recently, more attention has been paid to the association between trace mineral intake and depression [10,11,12,13,14,15,16,17,18] because several trace minerals are involved in the underlying pathophysiology of depression [19,20,21,22]. However, relatively few studies have explored the association between trace mineral intake and mental disorders, and the results are also still controversial.

Thus, in this study we investigated the association between the intake of six minerals and mental disorders in a cross-sectional study among the Japanese working population.

## 2. Materials and Methods 

### 2.1. Study Design and Participants

We used data from the Eating Habit and Well-being (Eat-Well) study in Japanese workers [23]. Briefly, a questionnaire survey for workers was conducted between December 2013 and February 2014 in Shizuoka, Japan. The study participants were employees that belonged to 43 small- and medium-sized manufacturing companies, because approximately 85% of workers in Japan are employed by such companies [24]. An invitation and questionnaires were mailed to the company and distributed to the employees by the management department of each company. The employees who were willing to join the survey completed the anonymous self-administered questionnaire, including the food frequency questionnaire (FFQ) [25]. Then, the questionnaires were submitted to the managers of their companies and mailed to the research team at the Hamamatsu University School of Medicine. All employees (i.e., full-time, part-time, fixed-term, and dispatched) were included; however, we excluded participants who lacked Japanese literacy skills. Further details of the study design and participants have been described elsewhere [23]. Informed consent was obtained via submission of the completed questionnaires. This study was carried out in accordance with the Declaration of Helsinki. The study protocol was approved by the Ethics Committee of the Hamamatsu University School of Medicine (no. 25-203).

### 2.2. Measurements

Kessler’s six-item psychological distress scale (K6) was used to detect depression and anxiety symptoms [26,27]. The Japanese version of the K6 was developed according to a standard translation/back-translation procedure and was determined to have a screening performance equivalent to those reported with the original English version [28]. Each item of the K6 was rated on a 5-point Likert scale from 0 to 4, therefore, the total score ranged from 0 to 24, with a higher score indicating more severe psychological distress [26,27]. Depression and anxiety symptoms were identified with a cut-off point of 13+ based on previous Western [27] and Asian [29,30] studies. When screening mood and anxiety disorders according to the Diagnostic and Statistical Manual of Mental Disorders, Fourth Edition (DSM-IV), the Japanese version of the K6 showed comparable screening ability to the Center for Epidemiologic Studies-Depression Scale (CES-D), and cut-off points of 4/5 to screen for mood/anxiety disorders and 12/13 to screen for severe mental illness were proposed [30].

The validated FFQ developed for Japanese adults was used to assess diet as an exposure variable [25]. Briefly, the FFQ includes 87 food items and asks about the usual consumption rates and portion sizes during the previous month. The consumption frequencies include five categories for beverages (1–3 times/month, 1–2 times/week, 3–4 times/week, 5–6 times/week, and once/day), and six categories for foods (those for beverages plus twice/day). The portion sizes include three categories for foods, and cups of drinks for beverages. Energy and mineral intake were calculated based on the Standard Tables of Food Composition in Japan [31], and then, energy-adjusted mineral intake was obtained by adjusting the total energy intake using a nutrient residual model [32]. Further details of the dietary assessment are described elsewhere [23,25].

Data on smoking, alcohol drinking, work schedule (i.e., shift working or day working), medications for hypertension, hyperlipidemia, and diabetes, and body mass index (BMI; calculated with body weight (kg) divided by square root of height (m^2^)) were obtained by a self-administered questionnaire.

### 2.3. Statistical Analysis

A total of 2382 participants were included in the database of the Eat-Well study. Among the 2369 people aged 18–79 years with sex recorded, 2159 people completed the FFQ with a total energy intake ≥500 kcal/day or ≤4000 kcal/day. Participants with missing values for K6 items (*n* = 54), or with a history of depression (*n* = 16) were excluded, thus, a total of 2089 individuals were included in the final analyses.

Participant characteristics across the K6 score (cut-off: 12/13) were analyzed with the chi-square test for categorical variables and *t*-test for continuous variables. A multiple logistic regression analysis was performed to obtain the odds ratio (OR) and 95% confidence interval (CI) using depression and anxiety symptoms as the dependent variables, and quartiles of intake of six minerals as independent variables using the highest quartile as a reference. Age and sex were adjusted in Model 1; smoking, alcohol drinking, BMI, work schedule, and intake of folic acid, vitamin C, B_6_, B_12_, and PUFA were further adjusted in Model 2; and medications for hypertension, hyperlipidemia, and diabetes were further adjusted in Model 3 as potential confounding variables. As the intake of each mineral was highly correlated to the others, we did not adjust for the intake of each mineral to avoid multicollinearity in the statistical model. Trend was tested by multiple logistic regression analysis using quartiles of mineral intake as the continuous variable. Then, the dietary intake of each mineral was classified as high or low using each median. The association between depression and anxiety symptoms and high or low mineral intake was also evaluated by multiple logistic regression analysis using the combination of high intake as a reference. Missing data for exposure variables were excluded from each analysis.

The statistical analyses were conducted with IBM SPSS Statistics 25 (IBM, New York, NY, USA). All tests of significance were 2-tailed, with differences with a *p*-value < 0.05 considered significant.

## 3. Results

The prevalence of depression and anxiety symptoms identified by a K6 score of 13+ was 6.9% among the 2089 participants, 8.1% among 1453 men and 4.2% among 636 women. Table 1 shows the characteristics of the participants across the K6 score cut-off of 12/13. Participants with depression and anxiety symptoms (K6, 13+) were more likely to be younger and to have a lower dietary intake of minerals and vitamins. However, BMI, medications for hypertension, hyperlipidemia, and diabetes, smoking, alcohol drinking, shift working, and total energy and PUFA intake were not associated with depression and anxiety symptoms.

Table 2 shows the OR and 95% CI for depression and anxiety symptoms according to the quartiles of dietary mineral intake. The lowest quartiles of zinc, copper, and manganese intake showed a statistically high age- and sex-adjusted OR compared to the highest quartiles. When smoking, alcohol drinking, BMI, work schedule, and intakes of folic acid, vitamin C, B_6_, B_12_, and PUFA were further adjusted for in Model 2, all associations between depression and anxiety symptoms and zinc, copper, and manganese intake remained statistically significant (Model 2: zinc: OR, 1.91, 95% CI, 1.05–3.49; copper: OR, 2.36, 95% CI, 1.23–4.55; manganese: OR, 2.13, 95% CI, 1.02–4.43). When further adjustments were made in Model 3 for medications for hypertension, hyperlipidemia, and diabetes, OR for copper intake remained significant, but those for zinc and manganese intakes were slightly reduced and showed marginal significance (Model 3: zinc: OR, 1.66, 95% CI, 0.89–3.09; copper: OR, 2.35, 95% CI, 1.20–4.62; manganese: OR, 1.98, 95% CI, 0.93–4.24). Furthermore, the trend analyses for the association between depression and anxiety symptoms and zinc, copper, and manganese intake were also statistically significant in all models. When the analyses were stratified by sex (Supplemental Tables S1 and S2), the results were not substantially changed, although some results did not reach statistical significance, and the ORs were higher in women than in men in Model 1. After further adjustments, the multivariable-adjusted ORs of zinc and manganese intake tended to be lower than the age- and sex-adjusted ORs in men, and the multivariable-adjusted ORs for copper and manganese intakes tended to be higher than the age- and sex-adjusted ORs in women. When the analyses were stratified by age (Supplemental Tables S3 and S4), the results did not change substantially, although the multivariable-adjusted OR for zinc intake showed an insignificant association with depression and anxiety symptoms in participants aged ≥40 years. In contrast, there were no statistically significant associations between depression and anxiety symptoms and calcium, magnesium, or iron intake in the multivariable-adjusted models.

**Figure 1:**
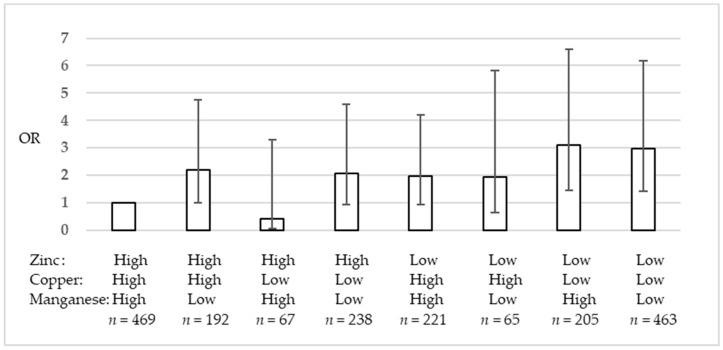
Odds ratios and 95% confidence intervals for depression and anxiety symptoms according to high or low zinc, copper, and manganese intake.

The intakes of the various minerals were broadly highly correlated among each other, with the exception of manganese intake (Supplemental Table S5). Figure 1 and Supplemental Table S6 show the ORs and 95% CIs for depression and anxiety symptoms according to the high or low intake of zinc, copper, and manganese, which showed significant associations in Table 2. Using high zinc, high copper, and high manganese intakes as reference, low zinc, low copper, and high manganese intake (Model 3: OR, 3.06, 95% CI, 1.41–6.61), and low zinc, low copper, and low manganese intake (Model 3: OR, 2.71, 95% CI, 1.29–5.73) showed statistically high ORs.

## 4. Discussion

This cross-sectional study in Japanese workers revealed that low intake of zinc, copper, and manganese was associated with depression and anxiety symptoms. The inverse association was not statistically significant for calcium, magnesium, and iron intakes. Furthermore, the combination analysis of high or low intake of zinc, copper, and manganese showed that low zinc and low copper intake, even with low or high manganese intake, had 3 times higher ORs for depression and anxiety symptoms compared to high zinc, high copper, and high manganese intake.

To date, several cross-sectional studies and a few cohort and intervention studies have reported on the association between trace mineral intake and depression; however, the findings are still controversial [10,11,12,13,14,15,16,17,18]. Among these, the association between zinc intake and depression has been most explored [10,11,12,13,14,15,16,17,18]. We observed an inverse association between zinc intake and depression and anxiety symptoms, which was broadly consistent with the results from the US [10,11] and Japan [12,13]. In addition, the inverse association between zinc intake and depression and anxiety symptoms was evident in women in the present study, in line with some previous studies [11,13]. A previous meta-analysis pointed out that preceding studies that investigated the association between zinc intake and depression used varying methodologies; however, the inverse association did not differ according to study design (cross-sectional or cohort studies), continent (America, Europe, or Asia), depression assessment methods (CES-D and others), or dietary assessment methods (FFQ and others) [16]. 

To the best of our knowledge, the association between copper [10,13] and manganese [13,14] intake and depression has rarely been investigated and remains controversial. The inverse association between copper intake and depression observed in the present study was broadly similar to the findings of previous studies [10,13]. In addition, we observed an inverse association between manganese intake and depression. Two previous Japanese studies investigating the association between manganese intake and depression showed controversial results: one study observed an inverse association among pregnant women [14], and one study observed an insignificant association among both sexes [13].

Because the intake of various minerals tends to be highly correlated, we assessed the combination of high or low intake of zinc, copper, and manganese, instead of adjusting for the intake of each mineral in the statistical analyses. The results showed a 3 times higher risk of depression and anxiety symptoms when the intake of zinc and copper was simultaneously low. The results indicate that low intake of these minerals may affect the risk for depression and anxiety symptoms in an additive manner.

Zinc is one of the most plausible dietary factors underlying the pathophysiological mechanisms of depression, although the pathophysiological mechanisms of depression are still unclear and are thought to involve a multi-factorial process [19,20,21,22]. The antidepressant effects of zinc have been explored in animal models of depression [19]. In clinical studies, the blood concentration of zinc in depressed subjects was low compared to that in non-depressed control subjects [33]. Evidence from intervention studies suggests potential benefits of zinc supplementation as an adjunctive therapy to antidepressant drug treatment; however, there is less clear evidence regarding the effect of zinc supplementation in the prevention of depressive symptoms in healthy subjects [15]. Several potential mechanisms underlying the association between zinc and depression have been proposed, and may involve neurotransmitters, endocrine, neurogenesis, oxidative stress pathways, and inflammation [19,20,21,22,34,35].

With regard to neurotransmitters, different minerals are thought to link to the different mechanisms [19,20]. Zinc, magnesium, iron, copper, and manganese are involved in the glutamatergic system; zinc, magnesium, calcium, chromium, lithium, copper, and selenium are involved in the monoaminergic system; zinc, magnesium, copper, and manganese are involved in the GABAergic (GABA: gamma-aminobutyric acid) system; zinc and magnesium are involved in the limbic-hypothalamus-pituitary-adrenocortical (HPA) axis-mediated stress response; and copper, selenium, and manganese are involved in the immune system [19,20]. Mlyniec summarized that zinc, magnesium, selenium, iron, iodine, and chromium may be inversely associated with depression; copper and calcium may be positively associated with depression; and manganese may be inversely or positively associated via the neurotransmitter systems involved in the pathophysiology of depression [19,20].

Oxidative stress pathways may also underlie the pathophysiology of depression [21,22]. Oxidative stress occurs as a result of the production of excessive reactive oxygen products or a deficient anti-oxidant defense system [36]. Previous studies revealed that the total antioxidant capacity was lower in depressed patients than in controls, and the levels of enzymatic and non-enzymatic antioxidants, including the levels of serum paraoxonase, superoxide dismutase (SOD), uric acid, albumin, high-density lipoprotein cholesterol, and vitamin C, were lower in patients than in controls [21,22]. On the other hand, the levels of oxidative damage products, including malondialdehyde and 8-F_2_-isoprostaned levels, were significantly higher in patients than in controls [21,22]. Meanwhile, after antidepressant therapy, the levels of some antioxidants, including serum uric acid, albumin, and vitamin C levels, were significantly increased [22]. 

Among the enzymatic antioxidants, SOD is widely distributed in the mitochondrion, nucleus, cytoplasm, and extracellular spaces, and has a vital antioxidant role in human health [36]. SODs are a group of metal-containing enzymes (Cu, Zn-SOD, and Mn-SOD), which catalyze the conversion of superoxide anions to hydrogen peroxide [36]. Furthermore, zinc is an important factor for the proper functioning of multiple aspects of the immune system, dysfunction of which is one of the underlying causes of depression [37]. Zinc is also involved in regulating inflammatory cytokines, and its deficiency results in cell-mediated immune dysfunction [37]. Moreover, inflammation may induce free radical formation, and oxidative stress may in turn induce an inflammatory response [38].

This study has several limitations. First, the cross-sectional nature of this study limits the inference of causality. We cannot deny the possibility of reverse causality, such as depressive participants taking less minerals owing to reduced appetite, although participants who have a history of depression were excluded from the present analysis. Second, instead of clinical examination or the use of a conventional depression scale, such as the CES-D, the K6 score with a cut-off point of 12/13 was used to determine outcome variables. However, previous studies have demonstrated good screening performance of the K6 in identifying depression and anxiety symptoms [27,30]. Third, FFQ was used for dietary assessment. The semi-quantitative nature of the FFQ limits the quantitative assessment of dietary intake, although it can reflect usual dietary intake. In the present study, mean zinc, copper, and manganese intake in participants with a K6 score <13 was 7.8 mg, 1.2 mg, and 3.9 mg per day, respectively, and the intake in participants with a K6 score ≥13 was 7.4 mg, 1.1 mg, and 3.5 mg per day, respectively. Although direct comparisons cannot be made because of the semi-quantitative nature of the FFQ, it is possible that the mean zinc intake in this study is lower than the mean zinc intake of Japanese adults (zinc 8.1 mg and copper 1.1 mg, manganese intake was not shown) estimated by the dietary record in the National Health and Nutrition Survey in Japan, 2017 [39], and the Recommended Dietary Allowance (RDA) for Japanese people (zinc 10 mg, copper 0.9–1.0 mg, and manganese 4.0 mg for men, and zinc 8 mg, copper 0.7–0.8 mg, and manganese 3.5 mg for women; the values for manganese were “Adequate Intake” instead of RDA) [40]. Forth, although we adjusted for several potential confounders, including the intake of vitamins C, B_6_, B_12_, folic acid, and PUFA, and medications for hypertension, hyperlipidemia, and diabetes, we cannot rule out the possibility that the results were still confounded by unmeasured potential confounders, such as use of dietary supplements and/or other medications. However, the daily use of dietary mineral supplements is low in Japan. A previous Japanese study reported that only 4.2% of women and 1.5% of men among the community-living population took mineral supplements, and most of them were calcium supplements, followed by iron and magnesium supplements, that would contribute less to zinc, copper, and manganese intake [41].

This cross-sectional study supports the inverse association between dietary zinc, copper, and manganese intake and depression and anxiety symptoms, independent of other dietary, lifestyle, and occupational factors, in the Japanese working population. Furthermore, the simultaneous low intake of zinc and copper seems to have an additive effect on this association. Further cohort or intervention studies are required to investigate the role of dietary mineral intake on mental health.

## Figures and Tables

**Table 1:** Characteristics of study participants across Kessler’s six-item psychological distress scale (K6) score.

	K6 score ≥ 13 (*n* = 144)		K6 score < 13 (*n* = 1945)		*p*-Value
Age (years), mean, SD	38.6	10.7	43.7	12.2	<0.001
Men, %	81.3		68.7		0.002
Body mass index (kg/m^2^), mean, SD	22.5	3.7	22.6	5.8	0.930
Medications for					
Hypertension, %	8.7		10.4		0.526
Hyperlipidemia, %	5.8		7.8		0.404
Diabetes, %	2.2		3.3		0.493
Current smoker, %	32.6		28.3		0.266
Alcohol drinking, %	41.0		41.7		0.865
Shift work, %	15.3		12.3		0.298
Dietary intake					
Total energy (kcal), mean, SD	2039.5	605.4	1946.0	586.2	0.066
Calcium (mg), mean, SD	405.4	172.6	439.4	159.6	0.014
Magnesium (mg), mean, SD	218.7	52.0	233.1	51.1	0.001
Iron (mg), mean, SD	7.0	2.2	7.6	2.1	0.001
Zinc (mg), mean, SD	7.4	1.4	7.8	1.0	0.001
Copper (mg), mean, SD	1.1	0.2	1.2	0.2	<0.001
Manganese (mg), mean, SD	3.5	1.1	3.9	1.2	<0.001
Folic acids (mg), mean, SD	280.8	89.5	308.8	93.7	0.001
Vitamin C (mg), mean, SD	83.8	39.4	95.8	40.7	0.001
Vitamin B_6_ (mg), mean, SD	1.1	0.3	1.1	0.3	0.001
Vitamin B_12_ (mg), mean, SD	3.8	1.7	4.4	1.7	<0.001
PUFA (g), mean, SD	11.2	3.3	11.4	2.7	0.536

SD: Standard deviation; PUFA: polyunsaturated fatty acids. Differences were tested using the chi-square test for categorical variables and *t*-test for continuous variables.

**Table 2:** Odds ratios and 95% confidence intervals for depression and anxiety symptoms according to quartile of dietary mineral intake.

	Model 1			Model 2			Model 3		
Dietary Intake	OR	95% CI		OR	95% CI		OR	95% CI	
Calcium									
Quartile 1	1.26	0.77	2.07	1.05	0.58	1.89	0.99	0.54	1.80
Quartile 2	0.78	0.46	1.33	0.72	0.41	1.26	0.61	0.34	1.09
Quartile 3	0.88	0.52	1.49	0.83	0.48	1.42	0.74	0.42	1.29
Quartile 4	1.00			1.00			1.00		
*p* for trend	0.333			0.927			0.905		
Magnesium									
Quartile 1	1.18	0.71	1.97	0.76	0.33	1.75	0.80	0.34	1.90
Quartile 2	0.76	0.44	1.31	0.59	0.30	1.16	0.63	0.32	1.26
Quartile 3	0.90	0.53	1.54	0.76	0.42	1.36	0.68	0.36	1.27
Quartile 4	1.00			1.00			1.00		
*p* for trend	0.514			0.481			0.702		
Iron									
Quartile 1	1.45	0.87	2.41	1.39	0.57	3.40	1.67	0.66	4.23
Quartile 2	0.85	0.49	1.48	0.83	0.40	1.72	0.84	0.39	1.81
Quartile 3	0.91	0.53	1.57	0.88	0.46	1.65	0.92	0.47	1.78
Quartile 4	1.00			1.00			1.00		
*p* for trend	0.107			0.379			0.218		
Zinc									
Quartile 1	1.74	1.06	2.85	1.91	1.05	3.49	1.66	0.89	3.09
Quartile 2	1.27	0.76	2.12	1.31	0.75	2.29	1.28	0.72	2.28
Quartile 3	0.80	0.46	1.41	0.82	0.46	1.48	0.76	0.41	1.39
Quartile 4	1.00			1.00			1.00		
*p* for trend	0.005			0.008			0.029		
Copper									
Quartile 1	2.03	1.20	3.42	2.36	1.23	4.55	2.35	1.20	4.62
Quartile 2	1.25	0.71	2.18	1.39	0.74	2.62	1.42	0.74	2.72
Quartile 3	1.23	0.70	2.17	1.34	0.73	2.45	1.23	0.66	2.31
Quartile 4	1.00			1.00			1.00		
*p* for trend	0.006			0.007			0.007		
Manganese									
Quartile 1	1.67	1.00	2.76	2.13	1.02	4.43	1.98	0.93	4.24
Quartile 2	1.01	0.58	1.74	1.23	0.62	2.46	1.10	0.53	2.25
Quartile 3	0.95	0.54	1.67	1.06	0.57	1.98	1.01	0.53	1.93
Quartile 4	1.00			1.00			1.00		
*p* for trend	0.024			0.014			0.026		

OR: Odds ratio; CI: Confidence interval. Model 1: Adjusted for age and sex; Model 2: Further adjustment for smoking, alcohol drinking, body mass index, shift work, and intake of Vitamin C, B_6_, B_12_, folic acid, and PUFA; Model 3: Further adjustment for medications for hypertension, hyperlipidemia, and diabetes.

## Supplementary Materials

The following are available online at https://www.mdpi.com/2072-6643/11/4/847/s1: Table S1: Odds ratios and 95% confidence intervals for depression and anxiety symptoms according to quartile of dietary mineral intake in men; Table S2: Odds ratios and 95% confidence intervals for depression and anxiety symptoms according to quartile of dietary mineral intake in women; Table S3: Odds ratios and 95% confidence intervals for depression and anxiety symptoms according to quartile of dietary mineral intake in participants aged <40 years; Table S4: Odds ratios and 95% confidence intervals for depression and anxiety symptoms according to quartile of dietary mineral intake in participants aged ≥40 years; Table S5: Pearson’s correlation coefficients among six mineral intakes; Table S6: Odds ratios and 95% confidence intervals for depression and anxiety symptoms according to high or low zinc, copper, and manganese intake.
