# Supplementary material for: Low Zinc, Copper, and Manganese Intake is Associated with Depression and Anxiety Symptoms in the Japanese Working Population: Findings from the Eating Habit and Well-Being Study
Source: Nutrients. 2019 Apr 15;11(4):847. doi: 10.3390/nu11040847 (PMC6521019; doi:10.3390/nu11040847)
Supplement: Supplementary file 1 [file nutrients-11-00847-s001.zip › nutrients-483418-SI.docx]

**Supplemental Table S1.** Odds ratios and 95% confidence intervals for depression and anxiety symptoms according to quartile of dietary mineral intake in men.

|  | **Model 1** | | | **Model 2** | | | **Model 3** | | |
| --- | --- | --- | --- | --- | --- | --- | --- | --- | --- |
| **Dietary intake** | **OR** | **95% CI** | | **OR** | **95% CI** | | **OR** | **95% CI** | |
| Calcium |  |  |  |  |  |  |  |  |  |
| Quartile 1 | 1.03 | 0.60 | 1.75 | 0.83 | 0.44 | 1.57 | 0.81 | 0.43 | 1.56 |
| Quartile 2 | 0.72 | 0.40 | 1.30 | 0.66 | 0.36 | 1.23 | 0.57 | 0.30 | 1.08 |
| Quartile 3 | 0.66 | 0.35 | 1.25 | 0.63 | 0.33 | 1.22 | 0.61 | 0.31 | 1.18 |
| Quartile 4 | 1.00 |  |  | 1.00 |  |  | 1.00 |  |  |
| P for trend | 0.563 |  |  | 0.786 |  |  | 0.695 |  |  |
| Magnesium |  |  |  |  |  |  |  |  |  |
| Quartile 1 | 1.34 | 0.73 | 2.45 | 0.95 | 0.36 | 2.48 | 0.84 | 0.32 | 2.24 |
| Quartile 2 | 0.92 | 0.48 | 1.76 | 0.75 | 0.34 | 1.65 | 0.72 | 0.32 | 1.58 |
| Quartile 3 | 1.09 | 0.56 | 2.10 | 0.98 | 0.48 | 2.00 | 0.78 | 0.37 | 1.65 |
| Quartile 4 | 1.00 |  |  | 1.00 |  |  | 1.00 |  |  |
| P for trend | 0.319 |  |  | 0.790 |  |  | 0.791 |  |  |
| Iron |  |  |  |  |  |  |  |  |  |
| Quartile 1 | 1.46 | 0.81 | 2.64 | 1.53 | 0.55 | 4.23 | 1.63 | 0.57 | 4.62 |
| Quartile 2 | 0.96 | 0.51 | 1.82 | 0.99 | 0.43 | 2.27 | 0.96 | 0.40 | 2.26 |
| Quartile 3 | 0.88 | 0.44 | 1.74 | 0.89 | 0.41 | 1.91 | 0.90 | 0.41 | 2.00 |
| Quartile 4 | 1.00 |  |  | 1.00 |  |  | 1.00 |  |  |
| P for trend | 0.084 |  |  | 0.280 |  |  | 0.247 |  |  |
| Zinc |  |  |  |  |  |  |  |  |  |
| Quartile 1 | 1.45 | 0.82 | 2.55 | 1.52 | 0.76 | 3.02 | 1.28 | 0.63 | 2.59 |
| Quartile 2 | 1.31 | 0.73 | 2.37 | 1.31 | 0.69 | 2.48 | 1.23 | 0.64 | 2.36 |
| Quartile 3 | 0.81 | 0.43 | 1.55 | 0.80 | 0.41 | 1.57 | 0.71 | 0.35 | 1.41 |
| Quartile 4 | 1.00 |  |  | 1.00 |  |  | 1.00 |  |  |
| P for trend | 0.057 |  |  | 0.077 |  |  | 0.180 |  |  |
| Copper |  |  |  |  |  |  |  |  |  |
| Quartile 1 | 1.94 | 1.06 | 3.55 | 2.10 | 1.01 | 4.36 | 2.07 | 0.97 | 4.44 |
| Quartile 2 | 1.31 | 0.68 | 2.50 | 1.34 | 0.65 | 2.76 | 1.45 | 0.69 | 3.05 |
| Quartile 3 | 1.20 | 0.61 | 2.36 | 1.23 | 0.60 | 2.50 | 1.23 | 0.59 | 2.57 |
| Quartile 4 | 1.00 |  |  | 1.00 |  |  | 1.00 |  |  |
| P for trend | 0.016 |  |  | 0.027 |  |  | 0.036 |  |  |
| Manganese |  |  |  |  |  |  |  |  |  |
| Quartile 1 | 1.51 | 0.85 | 2.66 | 1.35 | 0.58 | 3.12 | 1.38 | 0.58 | 3.26 |
| Quartile 2 | 0.87 | 0.47 | 1.61 | 0.80 | 0.36 | 1.76 | 0.75 | 0.33 | 1.70 |
| Quartile 3 | 0.91 | 0.48 | 1.72 | 0.84 | 0.41 | 1.71 | 0.82 | 0.39 | 1.71 |
| Quartile 4 | 1.00 |  |  | 1.00 |  |  | 1.00 |  |  |
| P for trend | 0.089 |  |  | 0.219 |  |  | 0.196 |  |  |

OR, Odds ratio; CI, Confidence interval. Model 1: Adjusted for age; Model 2: Further adjustment for smoking, alcohol drinking, body mass index, shift work, and intakes of Vitamin C, B6, B12, folic acid, and PUFA; Model 3: Further adjustment for medications for hypertension, hyperlipidemia, and diabetes.

**Supplemental Table S2.** Odds ratios and 95% confidence intervals for depression and anxiety symptoms according to quartile of dietary mineral intake in women.

|  | **Model 1** | | | **Model 2** | | | **Model 3** | | |
| --- | --- | --- | --- | --- | --- | --- | --- | --- | --- |
| **Dietary intake** | **OR** | **95% CI** | | **OR** | **95% CI** | | **OR** | **95% CI** | |
| Calcium |  |  |  |  |  |  |  |  |  |
| Quartile 1 | 3.02 | 0.90 | 10.08 | 2.69 | 0.57 | 12.62 | 2.21 | 0.42 | 11.67 |
| Quartile 2 | 0.66 | 0.17 | 2.61 | 0.62 | 0.14 | 2.81 | 0.56 | 0.12 | 2.55 |
| Quartile 3 | 1.47 | 0.57 | 3.81 | 1.35 | 0.47 | 3.90 | 1.13 | 0.38 | 3.36 |
| Quartile 4 | 1.00 |  |  | 1.00 |  |  | 1.00 |  |  |
| P for trend | 0.315 |  |  | 0.552 |  |  | 0.792 |  |  |
| Magnesium |  |  |  |  |  |  |  |  |  |
| Quartile 1 | 1.07 | 0.31 | 3.66 | 0.37 | 0.05 | 2.67 | 0.56 | 0.07 | 4.47 |
| Quartile 2 | 0.42 | 0.13 | 1.40 | 0.20 | 0.04 | 0.99 | 0.27 | 0.05 | 1.42 |
| Quartile 3 | 0.64 | 0.24 | 1.72 | 0.37 | 0.11 | 1.26 | 0.41 | 0.11 | 1.48 |
| Quartile 4 | 1.00 |  |  | 1.00 |  |  | 1.00 |  |  |
| P for trend | 0.536 |  |  | 0.203 |  |  | 0.436 |  |  |
| Iron |  |  |  |  |  |  |  |  |  |
| Quartile 1 | 1.75 | 0.55 | 5.62 | 0.99 | 0.13 | 7.58 | 1.83 | 0.22 | 15.40 |
| Quartile 2 | 0.36 | 0.08 | 1.69 | 0.25 | 0.04 | 1.70 | 0.19 | 0.02 | 2.13 |
| Quartile 3 | 1.11 | 0.43 | 2.83 | 0.81 | 0.23 | 2.84 | 1.00 | 0.27 | 3.75 |
| Quartile 4 | 1.00 |  |  | 1.00 |  |  | 1.00 |  |  |
| P for trend | 0.903 |  |  | 0.594 |  |  | 0.974 |  |  |
| Zinc |  |  |  |  |  |  |  |  |  |
| Quartile 1 | 4.38 | 1.57 | 12.22 | 4.28 | 1.16 | 15.77 | 4.39 | 1.08 | 17.84 |
| Quartile 2 | 1.02 | 0.32 | 3.28 | 0.98 | 0.27 | 3.64 | 1.25 | 0.32 | 4.92 |
| Quartile 3 | 0.68 | 0.20 | 2.34 | 0.83 | 0.22 | 3.07 | 0.92 | 0.24 | 3.52 |
| Quartile 4 | 1.00 |  |  | 1.00 |  |  | 1.00 |  |  |
| P for trend | 0.011 |  |  | 0.045 |  |  | 0.049 |  |  |
| Copper |  |  |  |  |  |  |  |  |  |
| Quartile 1 | 2.95 | 0.92 | 9.39 | 5.29 | 1.02 | 27.47 | 5.45 | 1.04 | 28.65 |
| Quartile 2 | 0.95 | 0.28 | 3.16 | 1.44 | 0.32 | 6.44 | 1.33 | 0.27 | 6.48 |
| Quartile 3 | 1.54 | 0.53 | 4.46 | 2.22 | 0.61 | 8.12 | 1.74 | 0.44 | 6.83 |
| Quartile 4 | 1.00 |  |  | 1.00 |  |  | 1.00 |  |  |
| P for trend | 0.186 |  |  | 0.110 |  |  | 0.074 |  |  |
| Manganese |  |  |  |  |  |  |  |  |  |
| Quartile 1 | 2.31 | 0.75 | 7.11 | 5.51 | 1.10 | 27.69 | 4.64 | 0.85 | 25.37 |
| Quartile 2 | 1.70 | 0.53 | 5.43 | 2.81 | 0.67 | 11.78 | 2.78 | 0.62 | 12.48 |
| Quartile 3 | 1.15 | 0.33 | 3.99 | 1.82 | 0.45 | 7.43 | 1.80 | 0.43 | 7.57 |
| Quartile 4 | 1.00 |  |  | 1.00 |  |  | 1.00 |  |  |
| P for trend | 0.111 |  |  | 0.032 |  |  | 0.066 |  |  |

OR, Odds ratio; CI, Confidence interval. Model 1: Adjusted for age; Model 2: Further adjustment for smoking, alcohol drinking, body mass index, shift work, and intakes of Vitamin C, B6, B12, folic acid, and PUFA; Model 3: Further adjustment for medications for hypertension, hyperlipidemia, and diabetes.

**Supplemental Table S3.** Odds ratios and 95% confidence intervals for depression and anxiety symptoms according to quartile of dietary mineral intake in participants aged <40 years.

|  | **Model 1** | | | **Model 2** | | | **Model 3** | | |
| --- | --- | --- | --- | --- | --- | --- | --- | --- | --- |
| **Dietary intake** | **OR** | **95% CI** | | **OR** | **95% CI** | | **OR** | **95% CI** | |
| Calcium |  |  |  |  |  |  |  |  |  |
| Quartile 1 | 1.03 | 0.52 | 2.04 | 1.07 | 0.46 | 2.45 | 1.14 | 0.49 | 2.65 |
| Quartile 2 | 0.62 | 0.30 | 1.30 | 0.64 | 0.29 | 1.41 | 0.58 | 0.26 | 1.30 |
| Quartile 3 | 0.87 | 0.43 | 1.76 | 0.90 | 0.43 | 1.86 | 0.87 | 0.41 | 1.83 |
| Quartile 4 | 1.00 |  |  | 1.00 |  |  | 1.00 |  |  |
| P for trend | 0.973 |  |  | 0.990 |  |  | 0.922 |  |  |
| Magnesium |  |  |  |  |  |  |  |  |  |
| Quartile 1 | 0.63 | 0.32 | 1.23 | 0.42 | 0.13 | 1.30 | 0.44 | 0.14 | 1.41 |
| Quartile 2 | 0.52 | 0.26 | 1.05 | 0.40 | 0.16 | 0.99 | 0.42 | 0.17 | 1.05 |
| Quartile 3 | 0.57 | 0.27 | 1.18 | 0.47 | 0.21 | 1.06 | 0.42 | 0.18 | 0.98 |
| Quartile 4 | 1.00 |  |  | 1.00 |  |  | 1.00 |  |  |
| P for trend | 0.284 |  |  | 0.206 |  |  | 0.288 |  |  |
| Iron |  |  |  |  |  |  |  |  |  |
| Quartile 1 | 0.89 | 0.45 | 1.76 | 0.94 | 0.28 | 3.20 | 1.16 | 0.32 | 4.18 |
| Quartile 2 | 0.65 | 0.31 | 1.34 | 0.65 | 0.24 | 1.76 | 0.81 | 0.29 | 2.27 |
| Quartile 3 | 0.83 | 0.41 | 1.70 | 0.80 | 0.34 | 1.90 | 0.87 | 0.36 | 2.14 |
| Quartile 4 | 1.00 |  |  | 1.00 |  |  | 1.00 |  |  |
| P for trend | 0.727 |  |  | 0.956 |  |  | 0.754 |  |  |
| Zinc |  |  |  |  |  |  |  |  |  |
| Quartile 1 | 1.61 | 0.82 | 3.16 | 2.65 | 1.11 | 6.32 | 2.84 | 1.17 | 6.92 |
| Quartile 2 | 1.25 | 0.63 | 2.47 | 1.70 | 0.79 | 3.66 | 1.78 | 0.80 | 3.95 |
| Quartile 3 | 0.77 | 0.36 | 1.65 | 0.98 | 0.43 | 2.19 | 0.95 | 0.41 | 2.20 |
| Quartile 4 | 1.00 |  |  | 1.00 |  |  | 1.00 |  |  |
| P for trend | 0.064 |  |  | 0.010 |  |  | 0.006 |  |  |
| Copper |  |  |  |  |  |  |  |  |  |
| Quartile 1 | 1.45 | 0.72 | 2.93 | 1.70 | 0.69 | 4.21 | 1.63 | 0.66 | 4.04 |
| Quartile 2 | 0.87 | 0.41 | 1.86 | 0.97 | 0.40 | 2.32 | 0.98 | 0.41 | 2.35 |
| Quartile 3 | 1.38 | 0.67 | 2.86 | 1.56 | 0.70 | 3.44 | 1.40 | 0.62 | 3.12 |
| Quartile 4 | 1.00 |  |  | 1.00 |  |  | 1.00 |  |  |
| P for trend | 0.506 |  |  | 0.438 |  |  | 0.419 |  |  |
| Manganese |  |  |  |  |  |  |  |  |  |
| Quartile 1 | 1.59 | 0.77 | 3.26 | 2.07 | 0.74 | 5.73 | 2.17 | 0.76 | 6.20 |
| Quartile 2 | 1.01 | 0.47 | 2.20 | 1.33 | 0.51 | 3.46 | 1.46 | 0.55 | 3.90 |
| Quartile 3 | 1.04 | 0.46 | 2.38 | 1.20 | 0.48 | 2.99 | 1.28 | 0.50 | 3.24 |
| Quartile 4 | 1.00 |  |  | 1.00 |  |  | 1.00 |  |  |
| P for trend | 0.140 |  |  | 0.107 |  |  | 0.107 |  |  |

OR, Odds ratio; CI, Confidence interval. Model 1: Adjusted for sex; Model 2: Further adjustment for smoking, alcohol drinking, body mass index, shift work, and intakes of Vitamin C, B6, B12, folic acid, and PUFA; Model 3: Further adjustment for medications for hypertension, hyperlipidemia, and diabetes.

**Supplemental Table S4.** Odds ratios and 95% confidence intervals for depression and anxiety symptoms according to quartile of dietary mineral intake in participants aged ≥40 years.

|  | **Model 1** | | | **Model 2** | | | **Model 3** | | |
| --- | --- | --- | --- | --- | --- | --- | --- | --- | --- |
| **Dietary intake** | **OR** | **95% CI** | | **OR** | **95% CI** | | **OR** | **95% CI** | |
| Calcium |  |  |  |  |  |  |  |  |  |
| Quartile 1 | 1.74 | 0.86 | 3.52 | 0.94 | 0.40 | 2.20 | 0.76 | 0.31 | 1.83 |
| Quartile 2 | 1.08 | 0.51 | 2.31 | 0.85 | 0.39 | 1.87 | 0.70 | 0.31 | 1.60 |
| Quartile 3 | 0.83 | 0.37 | 1.86 | 0.71 | 0.31 | 1.63 | 0.57 | 0.24 | 1.38 |
| Quartile 4 | 1.00 |  |  | 1.00 |  |  | 1.00 |  |  |
| P for trend | 0.079 |  |  | 0.978 |  |  | 0.671 |  |  |
| Magnesium |  |  |  |  |  |  |  |  |  |
| Quartile 1 | 2.93 | 1.38 | 6.20 | 1.52 | 0.45 | 5.10 | 1.59 | 0.43 | 5.81 |
| Quartile 2 | 1.26 | 0.54 | 2.92 | 0.89 | 0.32 | 2.44 | 1.02 | 0.35 | 2.96 |
| Quartile 3 | 1.59 | 0.72 | 3.49 | 1.28 | 0.54 | 3.03 | 1.25 | 0.48 | 3.25 |
| Quartile 4 | 1.00 |  |  | 1.00 |  |  | 1.00 |  |  |
| P for trend | 0.008 |  |  | 0.716 |  |  | 0.614 |  |  |
| Iron |  |  |  |  |  |  |  |  |  |
| Quartile 1 | 2.98 | 1.42 | 6.29 | 2.13 | 0.60 | 7.56 | 2.40 | 0.64 | 8.97 |
| Quartile 2 | 1.22 | 0.53 | 2.83 | 1.01 | 0.35 | 2.93 | 0.73 | 0.23 | 2.35 |
| Quartile 3 | 1.01 | 0.43 | 2.38 | 0.89 | 0.34 | 2.32 | 0.88 | 0.32 | 2.43 |
| Quartile 4 | 1.00 |  |  | 1.00 |  |  | 1.00 |  |  |
| P for trend | 0.001 |  |  | 0.170 |  |  | 0.141 |  |  |
| Zinc |  |  |  |  |  |  |  |  |  |
| Quartile 1 | 1.85 | 0.90 | 3.83 | 1.39 | 0.57 | 3.37 | 0.98 | 0.39 | 2.47 |
| Quartile 2 | 1.28 | 0.58 | 2.80 | 1.02 | 0.44 | 2.38 | 0.92 | 0.39 | 2.18 |
| Quartile 3 | 0.89 | 0.38 | 2.06 | 0.71 | 0.30 | 1.70 | 0.61 | 0.25 | 1.48 |
| Quartile 4 | 1.00 |  |  | 1.00 |  |  | 1.00 |  |  |
| P for trend | 0.045 |  |  | 0.267 |  |  | 0.728 |  |  |
| Copper |  |  |  |  |  |  |  |  |  |
| Quartile 1 | 3.57 | 1.63 | 7.81 | 4.22 | 1.58 | 11.24 | 4.52 | 1.59 | 12.84 |
| Quartile 2 | 2.14 | 0.93 | 4.89 | 2.34 | 0.91 | 6.02 | 2.45 | 0.89 | 6.72 |
| Quartile 3 | 1.02 | 0.40 | 2.61 | 1.04 | 0.39 | 2.77 | 0.90 | 0.30 | 2.66 |
| Quartile 4 | 1.00 |  |  | 1.00 |  |  | 1.00 |  |  |
| P for trend | <0.001 |  |  | 0.001 |  |  | <0.001 |  |  |
| Manganese |  |  |  |  |  |  |  |  |  |
| Quartile 1 | 2.34 | 1.17 | 4.67 | 2.73 | 0.98 | 7.64 | 2.32 | 0.79 | 6.84 |
| Quartile 2 | 1.19 | 0.56 | 2.56 | 1.35 | 0.50 | 3.62 | 0.97 | 0.34 | 2.80 |
| Quartile 3 | 0.93 | 0.43 | 2.02 | 0.95 | 0.40 | 2.27 | 0.82 | 0.33 | 2.03 |
| Quartile 4 | 1.00 |  |  | 1.00 |  |  | 1.00 |  |  |
| P for trend | 0.010 |  |  | 0.019 |  |  | 0.043 |  |  |

OR, Odds ratio; CI, Confidence interval. Model 1: Adjusted for sex; Model 2: Further adjustment for smoking, alcohol drinking, body mass index, shift work, and intakes of Vitamin C, B6, B12, folic acid, and PUFA; Model 3: Further adjustment for medications for hypertension, hyperlipidemia, and diabetes.

**Supplemental Table S5.** Pearson's correlation coefficients among six mineral intakes.

|  | **Magnesium** | **Iron** | **Zinc** | **Copper** | **Manganese** |
| --- | --- | --- | --- | --- | --- |
| Calcium | 0.719*** | 0.599*** | 0.547*** | 0.376*** | 0.102*** |
| Magnesium | - | 0.890*** | 0.581*** | 0.653*** | 0.266*** |
| Iron | - | - | 0.592** | 0.668*** | 0.425*** |
| Zinc | - | - | - | 0.650*** | 0.119*** |
| Copper | - | - | - | - | 0.514*** |
|  | | | | | |

n=2,089, *** p<0.001.

**Supplemental Table S6.** Odds ratios and 95% confidence intervals for depression and anxiety symptoms according to high or low zinc, copper, and manganese intake.

| **Zinc** | **Copper** | **Manganese** | **Cases** | **OR** | **95% CI** | |
| --- | --- | --- | --- | --- | --- | --- |
| High | High | High | 469 | 1.00 |  |  |
| High | High | Low | 192 | 1.83 | 0.81 | 4.12 |
| High | Low | High | 67 | 0.41 | 0.05 | 3.20 |
| High | Low | Low | 238 | 2.09 | 0.93 | 4.68 |
| Low | High | High | 221 | 1.88 | 0.87 0.93 | 4.07 |
| Low | High | Low | 65 | 1.70 | 0.51 | 5.63 |
| Low | Low | High | 205 | 3.06 | 1.41 | 6.61 |
| Low | Low | Low | 463 | 2.71 | 1.29 | 5.73 |
| Participants, n=1,920. OR, Odds ratio; CI, Confidence interval. High intake includes Quartiles 3 and 4, and low intake includes Quartiles 1 and 2. Age, sex, smoking, alcohol drinking, body mass index, shift work, intakes of Vitamin C, B_6_, B_12_, folic acid, and PUFA, and medications for hypertension, hyperlipidemia, and diabetes were adjusted for. | | | | | | |
